# The Struggle to Belong for Underrepresented Medical Students: A Narrative Review

**DOI:** 10.5334/pme.1873

**Published:** 2025-11-14

**Authors:** Victoria Luong, Paula Cameron, Megan E. L. Brown, Sarah Burm, Jordin Fletcher, Olga Kits, Anna MacLeod, Robin Parker, Rola Ajjawi

**Affiliations:** 1 Continuing Professional Development and Medical Education, Faculty of Medicine, Dalhousie University, Halifax, Nova Scotia, Canada; 2 Newcastle University, United Kingdom; 3 Dalhousie University, Halifax, Nova Scotia, Canada; 4 Research Methods Unit, Research, Innovation & Discovery, Nova Scotia Health, Halifax, Nova Scotia, Canada; 5 WK Kellogg Health Sciences Library, Dalhousie University, Halifax, Nova Scotia, Canada; 6 University of British Columbia, Vancouver, British Columbia, Canada

## Abstract

**Introduction:**

The concept of belonging (i.e., the feeling of connectedness with the people and environment around you) may, at first glance, seem straightforward and aspirational. However, there is a dark side to belonging when it becomes a driver to conform to unspoken and socially enforced group expectations. Applying critical sociological theory of the personal and political of belonging to undergraduate medical education, we asked: what are the experiences of belonging for underrepresented medical students?

**Methods:**

We conducted a narrative review of 16 qualitative research articles. We included papers published up to October 2024 that studied underrepresented medical students, defined as students who have been historically marginalized in medicine. Analysis involved interpreting students’ experiences of personal and political belonging and the consequences of the interplay between these two dimensions.

**Results:**

Underrepresented medical students perceived a marked division between themselves and their peers—who they described as ‘a different breed.’ They saw medicine as an ‘exclusive club’ where their differences were highly visible and felt excluded from activities that were considered foundational for peer bonding. A lack of understanding from, and commonalities with, other students, as well as experiences with prejudice and social exclusion within the curriculum and from educators, negatively impacted students’ ability to feel at home in medical school.

**Discussion:**

Our review highlights underrepresented medical students’ struggle to belong in medical school. This struggle is not simply a matter of personal connection; it is rooted in systemic oppression and can have profoundly harmful impacts, ultimately influencing professional identity formation, choice of specialty, and well-being.

## Introduction

The renewed turn to social justice in medical education has fuelled extensive research into the lived experiences of underrepresented students in medical school. This includes a burgeoning body of qualitative work seeking to explain, in-depth, what it is like to be an underrepresented learner in an institution steeped in privilege. In order to make sense of this developing body of literature, we previously conducted a meta-ethnographic synthesis of qualitative studies on the experiences of underrepresented students during undergraduate medical education. We found that underrepresented medical students performed much additional work to inhabit medical school environments and to embody the ideal doctor [1]. This prompted us to wonder how such exclusion might influence students’ sense of belonging. Therefore, in this narrative review, we revisited the studies from our meta-ethnography to investigate this cross-cutting element of underrepresented students’ stories.

Belonging, in the context of education, is commonly described as students’ feelings of connectedness with the people and places in their environment [2,3]. It involves feeling accepted, valued, and respected [3,4] and has been shown to be protective against stress and burnout [5,6,7] and to support academic success [2,3,8,9]. The need to belong may be particularly salient within the context of medical education, as the competitive nature of medical training increases the stakes of conformity [10,11]. After earning their spot through a rigorous process of elimination, students may feel an intense pressure to prove that they ‘belong’ in medicine and deserve to be seen as legitimate members of this elite profession—otherwise, they risk ostracization and rejection [12,13,14]. Proving that they belong includes passing their exams, but it also includes building the right relationships, speaking the right language, and demonstrating the right values [15].

We conducted this narrative review to examine underrepresented medical students’ experiences with belonging in medical school. We sought to understand the factors shaping their belonging, as well as the consequences of their struggle to belong in medical school.

### Theoretical framework

We turned to critical theories of belonging to analyze the experiences of underrepresented medical students. Yuval-Davis [16,17], a British sociologist studying immigration, differentiates between two concepts: *belonging* and the *politics of belonging*. Belonging (which we will call personal belonging, for clarity) describes an intimate emotional attachment towards a place (both physical and social space). It is a sense of feeling at home in a place. The political dimension of belonging pertains to the broader social and political processes that define who is included or excluded from a place. It involves the maintenance of boundaries, including the determination of what is involved in belonging, being a member of a community, and the roles specific social locations and specific narratives of identity play in belonging [16]. Healy suggests that there is a wide spectrum of ways people can struggle with belonging. In particular, he distinguishes between: *not-belonging*, which is a loss of a sense of belonging, and *unbelonging*, which is a rejection by those who have the power to grant membership belonging [18].

Belonging is complex to talk about, in part because what, exactly, students *belong to* is fluid and contextual. For example, students may have a sense of belonging to the profession (and becoming a medical doctor), even while they do not feel a sense of kinship with their peers. What is clear within the research literature is that belonging is neither a static, nor straightforward concept [13,19,20]. Therefore, in this paper, we conceptualize belonging as always being in negotiation and constituted in the interaction of people, spaces, places, and temporalities [21]. Based on this perspective, belonging is a process rather than a totalizing experience or destination—a process that is always situated and contingent, despite how we might talk about it and measure it.

Applying this critical, dynamic theoretical framework enabled us to analyse underrepresented students’ experiences of medical school conveyed through two analytical planes: the personal and political. We asked: how do underrepresented medical students narrate their experiences of belonging in medical school?

## Methods

The current study consists of a narrative review of a sub-set of papers from a meta-ethnography of underrepresented medical students’ experiences [1]. We chose a narrative approach to examine more closely, with our critical theoretical frame, the stories of how underrepresented students experience belonging in medical school [27].

In both the current study and the meta-ethnography, underrepresented medical students were defined as students who have been historically excluded from medicine [1]. We chose to keep this definition relatively broad to capture a wide range of social locations, while also limiting our search to groups that are likely to have commonalities in their experiences with belonging due to the historical and systemic barriers they have faced. Specifically, we searched for: racialized students, Indigenous students, disabled students, students from rural and/or low socioeconomic backgrounds, first generation (or first-in-family) medical students, and sexual orientation and gender identity minority students [1]. We selected studies using the following criteria: 1) qualitative study of any type, 2) medical school or undergraduate medical education, and 3) underrepresented medical students. The studies came from a range of sources: citation database searches in MEDLINE (Ovid) and Scopus, reference checking, and input from individuals with research and lived experience related to underrepresented students in medical school [1].

### Data generation: critical narrative review

From the 49 studies included in the meta-ethnography [1], we focused this narrative review on qualitative studies that explored the concept of belonging. We did not aim to be comprehensive in this search; rather, we sought to use a conceptual and theoretical lens to reexamine belonging in the qualitative investigations of underrepresented medical students’ experiences in medical school.

Four authors (PC, VL, MELB, OK) coded the 49 papers from the original review for themes and concepts. After this initial analysis, the code ‘belonging’ appeared in 13 papers. Two authors (VL and RA) retrieved these codes and evaluated, upon consensus, if the coded data reflected the definition of belonging that we selected for our narrative review. VL and RA then re-read each of the 49 papers for explicit reference to student belonging, to determine if any articles were missed in the original coding. Three additional papers were added at this stage. We included studies that related to belonging as defined by a sense of attachment or kinship to a place, which is negotiated by sociopolitical values, structures, and relationships. Rather than search for specific terms in the data, we were searching for concepts that reflected our definition of belonging; selected articles therefore often included the word ‘belonging,’ but also terms such as ‘fitting in’ and ‘feeling part of the group.’ Sixteen papers were included in this narrative review [15,22,23,24,25,26,27,28,29,30,31,32,33,34,35,36].

### Data analysis

VL and RA read each of the 16 papers independently, paying attention to participants’ quotes and researcher interpretations in relation to personal and political dimensions of belonging. The third category we analyzed was the intersection of the two dimensions, which we refer to below as the consequences of the struggle to belong. We also extracted contextual data about the studies including country of first author, research approach, overall sense of findings, and their reflexivity (see Table 1 for characteristics of the included papers and supplementary material for a full breakdown of included papers). VL then extracted relevant data from each paper and organized these into a table. The research team met via Microsoft Teams to draw together findings from the multiple studies and develop coherent interpretations of underrepresented medical students’ struggle to belong, and the ways they are excluded along systemic and structural lines. Team discussions focused on building a whole (i.e., a coherent narrative) from parts (i.e., the individual findings from each study), as well as comparing and contrasting themes among the different studies [37].

**Table 1:** Summary of article characteristics.


CATEGORY	NUMBER	ARTICLES

**Location**		

United States	4	[24,29,33,36]

United Kingdom	4	[23,26,30,31]

Canada	4	[15,25,34,35]

Australia	2	[22,32]

Netherlands	2	[27,28]

Underrepresented Group*		

Racial/Ethnic minority	9	[15,24,27,28,29,30,31,34,36]

Disabled	1	[23]

Black	3	[31,33,36]

First in Family	3	[22,32,35]

2SLGBTQ+**	3	[24,25,30]

Indigenous	3	[22,24,32]

Working class	2	[22,26]

**Methodologies**		

Qualitative study	9	[22,27,28,29,31,32,33,34,35]

Grounded theory	3	[24,25,36]

Phenomenography	1	[30]

Mixed methods	1	[15]

Participatory action research	1	[26]

Collaborative Autoethnography	1	[23]

**Methods**		

Interviews	12	[15,22,24,25,27,29,30,32,33,34,35,36]

Focus Group (FG)	2	[28,31]

Interview & arts-based (comics)	1	[26]

Interviews & written reflections	1	[23]


* This indicates the ‘focus’ student group(s) in each paper; multiple groups may be represented in each paper totaling more than 16. ** 2-Spirit, Lesbian, Gay, Bisexual, Transgender, and Queer.

### Reflexivity

Reflexivity can be defined as a “set of continuous, collaborative, and multifaceted practices through which researchers self-consciously critique, appraise, and evaluate how their subjectivity and context influence the research process” [38, p. 242]. In order to continuously recognize and account for our own subjectivity throughout this project, we engaged with reflexivity through individual and team reflections, discussions, and planned dedicated meetings at each level of data collection and analysis.

Our team was composed of individuals from diverse backgrounds (racial/ethnic backgrounds, disabilities, gender identities, and sexual orientations) and professional roles (medical students, graduate students, researchers, librarians, professors). Hence, we considered the multiple ways our intersecting and overlapping identities shaped our interpretations of the data and our approaches to understanding belonging in the medical education context. We continuously reflected, individually and as a team, through written and oral modes, on the complex interplay of our multiple identities, the stories of our own lives, and how these might inform our emotional reactions to the texts we were reading. This reflection allowed us to critically consider the influence of our backgrounds and experiences on the review and the research process. In a team reflexivity workshop, we read a poem considering unbelonging and discussed the ways our identities could simultaneously contribute to a sense of belonging and the struggle to belong in the context of medical schools. These experiences and reflections provided us with a better sense of how our subjectivity influenced our readings and interpretation of the data related to belonging in the literature.

## Results

### Characteristics of included studies

Sixteen papers were included in the final sample, with 221 participants’ perspectives being captured across all studies. Table 1 shows characteristics of the papers. All studies were qualitative and the majority utilised interviews. All were from the Global North. Student identities and characteristics such as socioeconomic status, ethnicity, disability, and being the first in family to be accepted in medicine were identified as boundaries for belonging. Three studies involved Indigenous students, but they were not the majority in any of the included studies [22,24,32]. Four studies used intersectionality as an explicit theoretical frame [26,28,30,32]. However, multi-dimensional demographic details were generally absent in these studies, and article conventions (including those focused on participant confidentiality) limited our ability to explore intersectional experiences [1]. A list of included papers with more information is available in supplementary material (Table 1).

### Dimensions of Belonging

**Figure 1:**
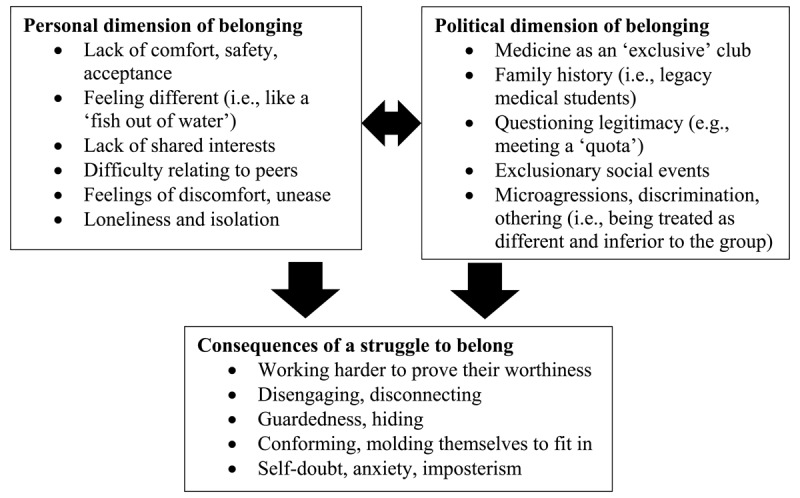
Personal and political dimensions of belonging in undergraduate medical education, and the consequences of a struggle to belong.

Medical students described both personal and political dimensions of belonging as they narrated their experiences in medical school. These findings are summarized in Figure 1. Students described feeling different from their peers and often lacked the comfort and safety that come with the sense that they belong. They recognized medicine as being an exclusive club that, by virtue of their identities, they did not belong to. They were victims of microaggressions and discrimination, which further impacted their belonging. Consequently, they struggled with the need to conform and often felt disengaged, anxious, and guarded.

### Personal dimension of belonging: being free to be themselves

Underrepresented medical students conceived of belonging as a feeling of comfort, safety, and acceptance within a group. When they belonged, they felt respected and ‘less compelled to think about or suppress aspects of their identities’ [24] (p. 334). It was as if they were embraced into a ‘family’— one in which they felt acknowledged and cared for:

[Belonging is the] ability to exist as their authentic, “true self” (P06) without “[having] to put on a certain face or certain personality [15] (p. 11)

Or being able to see an aspect of themselves in another:

…my attending was gay and … it’s kind of like the game recognize game feeling … [I felt] secure in a way [to be] more open in the way that I talk about things and in my mannerisms. [29] (p. 333)

However, students’ experiences of belonging tended to be fleeting. Underrepresented students often struggled with relating to other dominant group students [32] and described being a fish out of water [22,35], perceiving no ‘click’ between students of diverse backgrounds [27], and noting differences in ‘the language, the way you have to dress, [and] the way you have to speak’ [15, p. 627]. They did not share the same (generally expensive) hobbies with these students, such as skiing and travelling [15,35], and their financial challenges made them ‘never even [consider themselves] part at all of medical school people’ [26, p. 1079].

In addition, not belonging was associated with a sense of unease and discomfort in social situations where most people had ‘no understanding of you or those aspects of your identity’ [34, p. 542]. This struggle to belong ultimately led to loneliness and ‘a huge sense of isolation’ [22, p. 846]. This absence of shared representation and lived experience reflected by peers, teachers, and staff signaled to students that they are not supposed to be here—which we turn to next.

### Political dimension of belonging: the exclusive club

Underrepresented students perceived of medical school as an ‘exclusive’ club [22,26,15] in which only the privileged could feel like they belonged. Having a doctor in the family was a way many students gained legitimacy as members of that club [22], as long-established social networks rendered their place in medicine a given [15] ‘by virtue of how they grew up’ [34, p. 542]. Those who did not come from such families struggled to fit within these spaces:

…I regularly [socialise with medical students], but again it’s a bit hard to fit in I suppose. You know, when they’re talking about their father is a doctor, and what practice they’re at, and they have more connections when it comes to placements and things like that, whereas I have no idea because no-one’s in the medical field. [22] (p. 847)

This ease of belonging to medicine, for some students, can be contrasted with underrepresented students’ experiences of being questioned about their ‘merit’ and with ‘assumptions of intellectual inferiority’ [31]. Students mentioned how ‘people assume that you got here because you met a quota’ [31], which undermined their legitimacy at medical school:

… [the student was] rolling their eyes and saying “yeah, but all these quotas are letting you know people who shouldn’t be here, just because of positive, uh, discrimination.” [31] (p. 5)

Major milestones, such as orientation week or introduction days, carried messages about who belonged in medicine [27,34]. Social events often centered around a culture of alcohol consumption, which excluded some students from peer-bonding activities [30,34]. A lack of participation in activities such as these was seen as grounds for social exclusion:

… they are looking for someone who fits in their team. And if the team is made up of people who like to go skiing and drink beer, and you come in with your headscarf and you do not drink alcohol, you do not go skiing and you pray five times a day, then they say: “No, we do not want her.” [28] (p. 274)

Exclusionary practices were sometimes quite direct. Microaggressions, discrimination, and othering (i.e., being treated as outside of the dominant group in a way that is stigmatizing and dehumanizing) were common. Students described having jokes directed at them about joining the basketball team; being interrogated by campus security; and being repeatedly called by the wrong name [33]. Supervisors and assessors were often guilty of such discriminatory behavior, such as criticizing students’ clothing [29], which served to uphold and reinforce cultural norms and power hierarchies [25,28]. Furthermore, language was identified as a marker of difference, reflecting and reinforcing existing power structures. This occurred, for example, among students from Indigenous backgrounds speaking a type of English that was looked down upon by the more ‘polished’ and ‘clean cut’ dominant medical students [32].

In turn, this political exclusion influenced students’ personal belonging, as students were ‘made to feel so disillusioned and out of place on the course’ [31]. In the next section, we explore the impact that this struggle to belong had on underrepresented students.

### Consequences of the struggle to belong: conforming, imposterism and self-doubt

Sivananthajothy and colleagues [15] identified two key coping strategies in their work on belonging. Students either overcompensated, working even harder than their peers to prove their worth, or they withdrew and disengaged with opportunities and activities. In their study, they described how students worked harder to prove themselves worthy of being recognized as legitimate members of the group. This included, for example, effortfully seeking internships and networking opportunities that were not handed to them by virtue of coming from ‘a line of doctors’ [32] (p. 17).

Guardedness and hiding were commonly cited across the papers [24,30,31]. In one example, students reported hiding aspects of themselves to minimize their peers’ ‘discomfort’ [26]. Students described staying silent [29] and trying to blend in:

I try…to keep my head a little bit lower. In terms of like not being picked out, so not wearing anything too representative of both sides like I’m looking as white and as straight as possible at all times. [30] (p. 5)

They also turned to ‘fitting in’ to achieve peer acceptance—intentionally molding themselves to the group rather than being able to safely exist as they are:

[Fitting in is about] changing who you are… to feel accepted by the group that you’re with, but [not] being your true authentic self. [15] (p. 11)

The pressure to conform out of fear of exclusion meant that students expended additional energy and emotional labor thinking about ‘really dumb, small things like a scrunchy’ [24]. At the same time, it was often difficult to hide their differences, despite their best efforts. One student with a disability explained that they ‘tried to hide [their] differences and quirks…But, [found] it difficult to conform in the same way as [their] peers’ [23, p.4080]. Despite recognizing the need to ‘play the game,’ students yearned for the opportunity to exist as their ‘true’ selves:

I’ll play the game … But at a certain point … this is not how I want to practice medicine. I want to do it in a way that feels true to myself and aligned with my identity and not feeling like I have to hide certain parts of myself. [24] (p. 330)

Repeated experiences with lack of representation, discrimination, and social exclusion led students to question their worthiness to be in medical school [26] and to report hyperconsciousness and constant anxiety about their social place [33]. One student described this feeling of being an imposter as an amalgam of experiences that communicate that you do not belong:

I thought to myself, my school doesn’t support me, patients don’t like me, why do I belong in medicine? … it’s just this continuity of experiences where you don’t feel you belong [that makes] you question your position in medicine. [15] (p. 628)

Many students expressed ‘feelings of inadequacy, self-doubt, and ultimately, imposter syndrome’ [15] (p. 17) and it influenced their choice of specialty [24]. One student even mentioned how the struggle to belong led some of their peers to drop out of medical school entirely [28]. Consequently, it was difficult for students who felt like they did not belong to imagine themselves as future physicians, and to form their professional identities [36].

## Discussion

In this review, we explored how underrepresented students narrated their struggle to belong in medical school. Students described feeling disconnected from their peers, experiencing constant microaggressions, and being taunted as not deserving a place in medicine based on merit [33]. These constant threats to belonging led students to adopt strategies like overwork to prove themselves or to hide [15]. The feelings of inadequacy and self-doubt they experienced were harmful to their wellbeing, with the potential to profoundly influence their academic success and the trajectory of their careers in medicine [15,24]. Hence, our findings suggest that there is a mismatch between medical school claims for social justice (and indeed significant effort to widen access) and students’ lived experiences of support and respect during medical school [39,40].

This review explored both personal and political dimensions of belonging. On the one hand, narratives of personal belonging demonstrated that belonging (be-*longing*) is a deeply emotional concept. As Probyn writes, ‘individuals and groups are caught within wanting to belong, wanting to become, a process that is fueled by yearning rather than positing of identity as a stable state’ [40]. Underrepresented medical students longed for a liberating sense of belonging that allowed them to express their ‘authentic’ selves. What is clear from this narrative review, however, is that underrepresented students feel unable to show up as their authentic selves in the face of exclusionary standards of professionalism [41].

Other literature reviews have evaluated how belonging has been defined and measured in health professions education [42,43] and the impact a struggle to belong can have on learners’ performance and wellbeing [43]. However, our review illustrates how this problem disproportionally impacts underrepresented medical students. Many students will struggle with belonging at some point during their training [42,43]; but, when students enter medical school and are surrounded by people with considerable resources who look, speak, and act differently than they do, this may lead them to continually question whether they are really supposed to be there—whether they are ‘smart enough’ or ‘entitled’ enough to be a doctor [32].

In a recent study exploring the relationship between imposter phenomenon and gender-based discrimination, LaDonna et al. [14] introduced the *intruder paradox* (IPx) as a concept distinguishable from the imposter phenomenon (IP). While IP can describe struggles with belonging that are experienced by all types of trainees, especially in the early years of their training, IPx represents the externally imposed alienation of those who do not fit (or resist) the traditional norms and values that have come to represent the profession [14]. The authors describe IPx as a form of gaslighting, because individuals who may have an internal sense of belonging are led—through their experiences and interactions with others—to (re)question their abilities, authority, or legitimacy to claim their place in the field. In the current narrative review, we found that underrepresented students experienced not only IP, but IPx, which deeply impacted their belonging. Returning to the theoretical perspective of this paper, being marked as an intruder delegitimated students’ belonging (unbelonging), contributing to isolation and loneliness (not belonging), and reproducing inequitable power and social hierarchies.

Belonging results from the interplay of two intersecting dimensions: one that seeks belonging and one that has the power of ‘granting’ belonging. Therefore, centering belonging as an essential part of the medical school experience requires a nuanced approach. On the one hand, focusing solely on fostering fleeting and silo-ed personal feelings of belonging (e.g., through social activities outside medical spaces, or individual strategies such as stress management and structured feedback [44]) risks overlooking the systemic inequalities and power dynamics that shape students’ experiences (i.e., the politics of belonging). Previous authors have critiqued concepts like IP for locating the ‘problem’ uniquely in students themselves, rather than growing from deeper cultural foundations [14]. On the other hand, focusing only on the politics of belonging might deemphasize the personal and emotional impact of unbelonging, and how it is experienced by each student. As this work shows, the personal and political dimensions feed into each other as belonging is attempted and at times bestowed, from moment to moment. By challenging belonging as a stable, fixed category, the field can move beyond belonging as a ‘territorialized construct’ and the essentialism of identities [45].

One potential for enhancing belonging beyond identity lines is to develop opportunities for students to work together to co-create work that makes a difference. For example, students’ agency might be fostered through meaningful participation in community to co-create learning resources [46,47]. Acknowledging the preexisting financial barriers and additional workloads of these students, and compensating them for their work, can help mitigate the minority tax that imposes the burden of curricular social justice-related efforts on underrepresented students [48]. In addition, the number of experiences with microaggressions and discrimination reported in the studies we reviewed was concerning; we encourage ongoing efforts to provide faculty and staff with the tools to identify these instances and take action [49]. In our analysis, we found that safe spaces for underrepresented medical students to be themselves tended to be in short supply [24,29,30]. Students clearly wanted some respite from the constant need to conform, perform inauthenticity, and fit in with the dominant medical culture. In the absence of safe spaces within the medical school, students sought out other spaces such as Diversity, Equity, and Inclusion offices on campus, society spaces, and other informal spaces outside of university and clinical settings.

Researching the politics of belonging prompts thinking about difference, inclusion, and exclusion. Attending to the politics of belonging means paying attention to internalizations of forced constructions of self and identity, and dismantling the narrow norms and expectations of being a physician. Widening access to medicine is not enough; underrepresented medical students need to be accepted and respected for their unique identities and contributions to medicine. Students should be given opportunities to construct aspects of themselves in ways that define what matters to them in their medical practice and their humanity.

### Limitations

This narrative review had the following limitations: first, because it only included studies published in English, the study primarily represents the perspectives of students studying in English-speaking countries. Furthermore, while the review aimed to capture a broad spectrum of identities and social locations, a disproportionate number of studies focused on racialized students, which may have influenced the research findings. Included papers tended to orient towards the social and structural barriers to belonging, with a few exceptions that attended to the material [25,29]. Future research should examine the material ways in which students are excluded (e.g., through policies, physical spaces, clothing) alongside a look at broader intersecting identities (e.g., Indigenous students, neurodivergence, and postgraduate trainees). Finally, as noted above, primary studies seldom reported participant demographic details and data in a multi-dimensional manner, and an intersectional analysis was not possible.

## Conclusion

In this narrative review, we offer a critical theoretical explanation of belonging as a dynamic process, consisting of personal and political dimensions. The disproportionate struggle that underrepresented students face in developing a sense of belonging is not simply a matter of personal connection; it is rooted in power dynamics that shape social exclusion. The struggle to belong can have profoundly harmful impacts, including the erasure of aspects of self and feeling like an imposter, which can ultimately influence professional identity formation and well-being.

## Additional Files

The additional files for this article can be found as follows:

10.5334/pme.1873.s1 Supplementary Material 1. Complete search strategy.

10.5334/pme.1873.s2 Supplementary Material 2. Derivation of the narrative review sample from the published meta-ethnography.

10.5334/pme.1873.s3 Supplementary Material 3. Characteristics of included studies.

## References

[1] Cameron P, Fletcher J, Brown M, Parker R, Luong V, Kits O, et al. Labour upon labour: A best evidence medical education (BEME) meta-ethnography of underrepresented students’ experiences of medical school. Med Teach. Published ahead of print 2025. DOI: 10.1080/0142159X.2025.2540413 40802180

[2] Allen KA, Slaten C, Hong S, Lan Ma, Craig H, May F, et al. Belonging in higher education: a twenty-year systematic review. J Univ Teach Learn Pract. 2024;21(5):1–55. DOI: 10.53761/s2he6n66

[3] Strayhorn TL. College students’ sense of belonging: a key to educational success for all students. 2nd ed. New York: Routledge; 2019. DOI: 10.4324/9781315297293

[4] Baumeister RF, Leary MR. The need to belong: Desire for interpersonal attachments as a fundamental human motivation. Psychol Bull. 1995;117(3):497–529. DOI: 10.1037/0033-2909.117.3.497 7777651

[5] Neufeld A, Mossiere A, Malin G. Basic psychological needs, more than mindfulness and resilience, relate to medical student stress: a case for shifting the focus of wellness curricula. Med Teach. 2020;42(12):1401–12. DOI: 10.1080/0142159X.2020.1813876 33016810

[6] Puranitee P, Kaewpila W, Heeneman S, van Mook W, Busari JO. Promoting a sense of belonging, engagement, and collegiality to reduce burnout: a mixed methods study among undergraduate medical students in a non-Western, Asian context. BMC Med Educ. 2022;22(327):1–12. DOI: 10.1186/s12909-022-03380-0 35484548 PMC9047274

[7] Leep Hunderfund AN, Saberzadeh Ardestani B, Laughlin-Tommaso SK, Jordan BL, Melson VA, et al. Sense of belonging among medical students, residents, and fellows: associations with burnout, recruitment retention, and learning environment. Acad Med. 2025;100(2):191–202. DOI: 10.1097/ACM.0000000000005892 39348173

[8] Ryan RM, Deci EL. Self-Determination Theory and the facilitation of intrinsic motivation, social development, and well-being. Am Psychol. 2000;55(1):68–78. DOI: 10.1037/0003-066X.55.1.68 11392867

[9] Vivekananda-Schmidt P, Sandars J. Belongingness and its implications for undergraduate health professions education: a scoping review. Educ Prim Care. 2018;29(5):268–75. DOI: 10.1080/14739879.2018.1478677 30063879

[10] Stelling V, Andersen CA, Suarez DA, Nordhues HC, Hafferty FW, Beckman TJ, Sawatsky AP. Fitting in while standing out: professional identity formation, imposter syndrome, and burnout in early-career faculty physicians. Acad Med. 2023 Apr;98(4):514–520. DOI: 10.1097/ACM.0000000000005049 36512808

[11] Luong V, Shields C, Petrie A, Neumann K. Does personality matter? Perceptions and experiences of introverts and extraverts as general surgeons. Teach Learn Med. 2022;34(3):255–265. DOI: 10.1080/10401334.2021.1922284 34000927

[12] Bourgeois-Law G, Teunissen PW, Varpio L, Regehr G. Attitudes towards physicians requiring remediation: one-of-us or not-like-us? Acad Med. 2019;94:S36–S41. DOI: 10.1097/ACM.0000000000002896 31365392

[13] Gonzalez-Flores A, Clay W, Madzia J, Harry-Hernandez S, Henderson D. Our souls look back and wonder: reflections on belonging and being invisible in medicine. Ann Fam Med. 2023;21(Suppl 2):S106–S8. DOI: 10.1370/afm.2916 36849486 PMC9970682

[14] LaDonna KA, Cowley L, Field E, Ginsburg S, Watling C, Pack R. Introducing the intruder paradox: “It’s not the imposter syndrome, it’s you don’t want me in the field”. Med Educ; 2025. Publication ahead of print. DOI: 10.1111/medu.15741 PMC12438053 40509598

[15] Sivananthajothy P, Adel A, Afhami S, Castrogiovanni N, Osei-Tutu K, Brown A. Equity, diversity, and…exclusion? A national mixed methods study of “belonging” in Canadian undergraduate medical education. Adv Health Sci Educ Theory Pract. 2024;29(2):611–39. DOI: 10.1007/s10459-023-10265-4 37563338

[16] Yuval-Davis N. Belonging and the politics of belonging. Patterns Prejud. 2006;40(3):197–214. DOI: 10.1080/00313220600769331

[17] Yuval-Davis N. Power, Intersectionality and the politics of belonging. In: Harcourt W, editor. The Palgrave Handbook of gender and development. London: Palgrave Macmillan UK; 2016. pp. 367–81. DOI: 10.1007/978-1-137-38273-3_25

[18] Healy M. The other side of belonging. Stud Philos Educ. 2020;39(2):119–33. DOI: 10.1007/s11217-020-09701-4

[19] Poitevien P, Kas-Osoka O, Burns A, Kester Prakash L, Marbin J, Schwartz A, et al. Upholding our PROMISE: Increased representation is not enough to foster belonging in graduate medical education. Med Educ. 2025 Jun;59(6):630–639. DOI: 10.1111/medu.15546 39317675

[20] Roberts SE, Shea JA, Sellers M, Butler PD, Kelz RR. Pursuing a career in academic surgery among African American medical students. Am J Surg. 2020;219(4):598–603. DOI: 10.1016/j.amjsurg.2019.08.009 31470975

[21] Gravett K, Ajjawi R. Belonging as situated practice. Stud High Educ. 2022;47(7):1386–1396. DOI: 10.1080/03075079.2021.1894118

[22] Brosnan C, Southgate E, Outram S, et al. Experiences of medical students who are first in family to attend university. Med Educ. 2016;50(8):842–51. DOI: 10.1111/medu.12995 27402044

[23] Walker ER, Shaw SCK, Anderson JL. Dyspraxia in medical education: a collaborative autoethnography. Qual Rep. 2020;25(11):4072–93. DOI: 10.46743/2160-3715/2020.4352

[24] Bullock JL, Sukhera J, Del Pino-Jones A, Pino-Jones AD, Dyster TG, Ilgen JS, et al. ‘Yourself in all your forms’: A grounded theory exploration of identity safety in medical students. Med Educ. 2024;58(3):327–37. DOI: 10.1111/medu.15174 37517809

[25] Butler K, Yak A, Veltman A. “Progress in medicine is slower to happen”: qualitative insights into how trans and gender nonconforming medical students navigate cisnormative medical cultures at Canadian training programs. Acad Med. 2019;94(11):1757–65. DOI: 10.1097/ACM.0000000000002933 31397706

[26] Foreshew A, Al-Jawad M. An intersectional participatory action research approach to explore and address class elitism in medical education. Med Educ. 2022;56(11):1076–85. DOI: 10.1111/medu.14857 35718997

[27] Isik U, Wouters A, Croiset G, Kusurkar RA. “What kind of support do I need to be successful as an ethnic minority medical student?” A qualitative study. BMC Med Educ. 2021;21(6):1–12. DOI: 10.1186/s12909-020-02423-8 33402191 PMC7786944

[28] Isik U, Wouters A, Verdonk P, Croiset G, Kusurkar RA. “As an ethnic minority, you just have to work twice as hard.” Experiences and motivation of ethnic minority students in medical education. Perspect Med Educ. 2021;10(5):272–8. DOI: 10.1007/S40037-021-00679-4 34515955 PMC8505584

[29] Karasz A, Nemiroff S, Joo P, Blanco I, Fishman AY, Kelly MS, et al. A sense of belonging: perceptions of the medical school learning environment among URM and non-URM students. Teach Learn Med. 2024;36(5):566–76. DOI: 10.1080/10401334.2023.2232347 37450615

[30] Ly D, Chakrabarti R. ‘I’m looking as white and as straight as possible at all times’: a qualitative study exploring the intersectional experiences of BAME LGBTQ+ medical students in the UK. BMJ Open. 2024;14(8):1–10. DOI: 10.1136/bmjopen-2024-086346 PMC11337697 39160106

[31] Morrison N, Zaman T, Webster G, Sorinola O, Blackburn C. ‘Where are you really from?’: a qualitative study of racial microaggressions and the impact on medical students in the UK. BMJ Open. 2023;13(5):1–15. DOI: 10.1136/bmjopen-2022-069009 PMC10163503 37147091

[32] Southgate E, Brosnan C, Lempp H, Kelly B, Wright S, Outram S, et al. Travels in extreme social mobility: how first-in-family students find their way into and through medical education. Crit Stud Educ. 2017;58(2):242–60. DOI: 10.1080/17508487.2016.1263223

[33] Strayhorn TL. Exploring the role of race in black males’ sense of belonging in medical school: a qualitative pilot study. Med Sci Educ. 2020;30(4):1383–7. DOI: 10.1007/s40670-020-01103-y 34457805 PMC8368274

[34] van Buuren A, Yaseen W, Veinot P, Mylopoulos M, Law M. Later is too late: exploring student experiences of diversity and inclusion in medical school orientation. Med Teach. 2021;43(5):538–45. DOI: 10.1080/0142159X.2021.1874326 33529540

[35] Wright SR, Boyd VA, Okafor I, et al. ‘First in family’ experiences in a Canadian medical school: a critically reflexive study. Med Educ. 2023;57(10):980–90. DOI: 10.1111/medu.15116 37226410

[36] Wyatt TR, Rockich-Winston N, Taylor TR, White D. What does context have to do with anything? A study of professional identity formation in physician-trainees considered underrepresented in medicine. Acad Med. 2020;95(10):1587–93. DOI: 10.1097/ACM.0000000000003192 32079956

[37] Noblit GW, Hare RD. Meta-ethnography: Synthesizing qualitative studies. Newbury Park, CA: SAGE; 1988. DOI: 10.4135/9781412985000

[38] Olmos-Vega FM, Stalmeijer RE, Varpio L, Kahlke R. A practical guide to reflexivity in qualitative research: AMEE Guide No. 149. Med Teach. 2022;44(3):241–51. DOI: 10.1080/0142159X.2022.2057287 35389310

[39] O’Shea S. “Kids from here don’t go to uni”: Considering first in family students’ belonging and entitlement within the field of higher education in Australia. Eur J Educ. 2020;56(1):65–77. DOI: 10.1111/ejed.12434

[40] Probyn E. Outside belongings. Milton Park, Abingdon: Routledge; 1996.

[41] Jean DA, Jacobson CE, Rodriguez I, Vitous A, Kwakye G. The hidden burden: qualitative differences in how URiM students experience the clinical microenvironment. J Surg Educ. 2023;80(3):372–84. DOI: 10.1016/j.jsurg.2022.10.012 36372726

[42] Saberzadeh-Ardestani B, Hunderfund AN, Reed DA, Usher EL. Measuring sense of belonging among current and future physicians: a systematic review. Acad Med. 2025 Jul 28. Epub ahead of print. DOI: 10.1097/ACM.0000000000006199 40828170

[43] Vivekananda-Schmidt P, Sandars J. Belongingness and its implications for undergraduate health professions education: a scoping review. Educ Prim Care. 2018 Sep 3;29(5):268–75. DOI: 10.1080/14739879.2018.1478677 30063879

[44] Kristoffersson E, Boman J, Bitar A. Impostor phenomenon and its association with resilience in medical education – a questionnaire study among Swedish medical students. BMC Med Educ. 2024;24(782):1–10. DOI: 10.1186/s12909-024-05788-2 39030556 PMC11264822

[45] Antonsich M. Searching for belonging – an analytical framework. Geogr Compass. 2010;4(6):644–59. DOI: 10.1111/j.1749-8198.2009.00317.x

[46] Yemane L, Powell C, Edwards J, Shumba T, Alvarez A, Bandstra B, et al. Underrepresented in medicine trainees’ sense of belonging and professional identity formation after participation in the Leadership Education in Advancing Diversity Program. Acad Pediatr. 2025;25(1):1–9. DOI: 10.1016/j.acap.2024.08.003 39117029

[47] Rae VI, Smith SE, Hopkins SR, Tallentire VR. From corners to community: exploring medical students’ sense of belonging through co-creation in clinical learning. BMC Med Educ. 2024;24(474):1–10. DOI: 10.1186/s12909-024-05413-2 38689267 PMC11059736

[48] Betancourt RM, Baluchi D, Dortche K, Campbell KM, Rodríguez JE. Minority tax on medical students: a review of the literature and mitigation recommendations. Fam Med. 2024;56(3):169–75. DOI: 10.22454/FamMed.2024.268466 38467005 PMC11136630

[49] Anjorin O, Busari JO. Unpacking the social constructs of discrimination, othering, and belonging in medical schools. Teach Learn Med. 2024;36(5):660–8. DOI: 10.1080/10401334.2023.2230211 37424255

