# Supplementary material for: The Struggle to Belong for Underrepresented Medical Students: A Narrative Review
Source: Perspect Med Educ. 2025 Nov 14;14(1):826–36. doi: 10.5334/pme.1873 (PMC12617423; doi:10.5334/pme.1873)
Supplement: Supplementary Material 1. — Complete search strategy. [file pme-14-1-1873-s1.pdf]

Supplementary Methods 1: Complete search strategy

Ovid MEDLINE(R) ALL <1946 to May 04, 2023>

1 ((("semi-structured" or semistructured or unstructured or informal or "in-depth" or indepth or "face-to-face" or structured or guide) adj3 (interview* or discussion* or questionnaire*)) or (focus group* or qualitative or ethnograph* or fieldwork or "field work" or "key informant")).ti,ab. or interviews as topic/ or focus groups/ or narration/ or qualitative research/ 512026 [from O'Dea, Á., Stanley, M., Coote, S., & Robinson, K. (2021). Children and young people's experiences of living with developmental coordination disorder/dyspraxia: A systematic review and meta-ethnography of qualitative research. Plos one, 16(3), e0245738.]
2 ((("semi-structured" or semistructured or unstructured or informal or "in-depth" or indepth or "face-to-face" or structured or guide) adj3 (interview* or discussion* or questionnaire*)) or (focus group* or qualitative or ethnograph* or fieldwork or "field work" or "key informant" or phenomenolog* or autoethnograph*)).ti,ab,kf. or interviews as topic/ or focus groups/ or narration/ or qualitative research/
3 (qualitative or experience* or perception* or perspective* or interview* or focus group* or mixed methods or participant observation or transcript* or ethnograph* or phenomenol* or grounded theor* or grounded-theor* or purposive sample or lived experience* or narrative* or life experience* or life stor* or action research or thematic analysis or narrative analysis or field stud* or field-notes or videorecording).mp. [from https://libguides.sph.uth.tmc.edu/search_filters/ovid_medline_filters]
4 2 or 3
5 Schools, Medical/
6 Students, Medical/
7 Education, Medical, Undergraduate/
8 ((medical or medicine) adj2 (undergraduate* or school* or education or student*)).ti,ab,kf.
9 5 or 6 or 7 or 8
10 ((underrepresented or "under represented" or uim or urm or urim or minorit* or marginali* or racial* or disabilit* or disabled or dyslexi* or neurodivers* or neuro-divers* or adhd or autis* or "sexual orientation*" or "gender identit*" or "non-traditional" or nontraditional or mature or "first in family" or "previous career" or immigra* or refugee* or "foster care" or parent* or indigenous or native or aboriginal or american indian or african american* or bame or black* or latin* or hispanic* or SOGI or gay or trans or transgender* or nonconforming or non-binary or queer or lgbt* or two spirit or "2 spirit" or divers*) adj3 (student* or trainee* or learner*)).ti,ab,kf,kw.
11 4 and 9 and 10
