# Supplementary material for: The Struggle to Belong for Underrepresented Medical Students: A Narrative Review
Source: Perspect Med Educ. 2025 Nov 14;14(1):826–36. doi: 10.5334/pme.1873 (PMC12617423; doi:10.5334/pme.1873)

Supplementary Figure 1:

Derivation of the narrative review sample from the published meta-ethnography

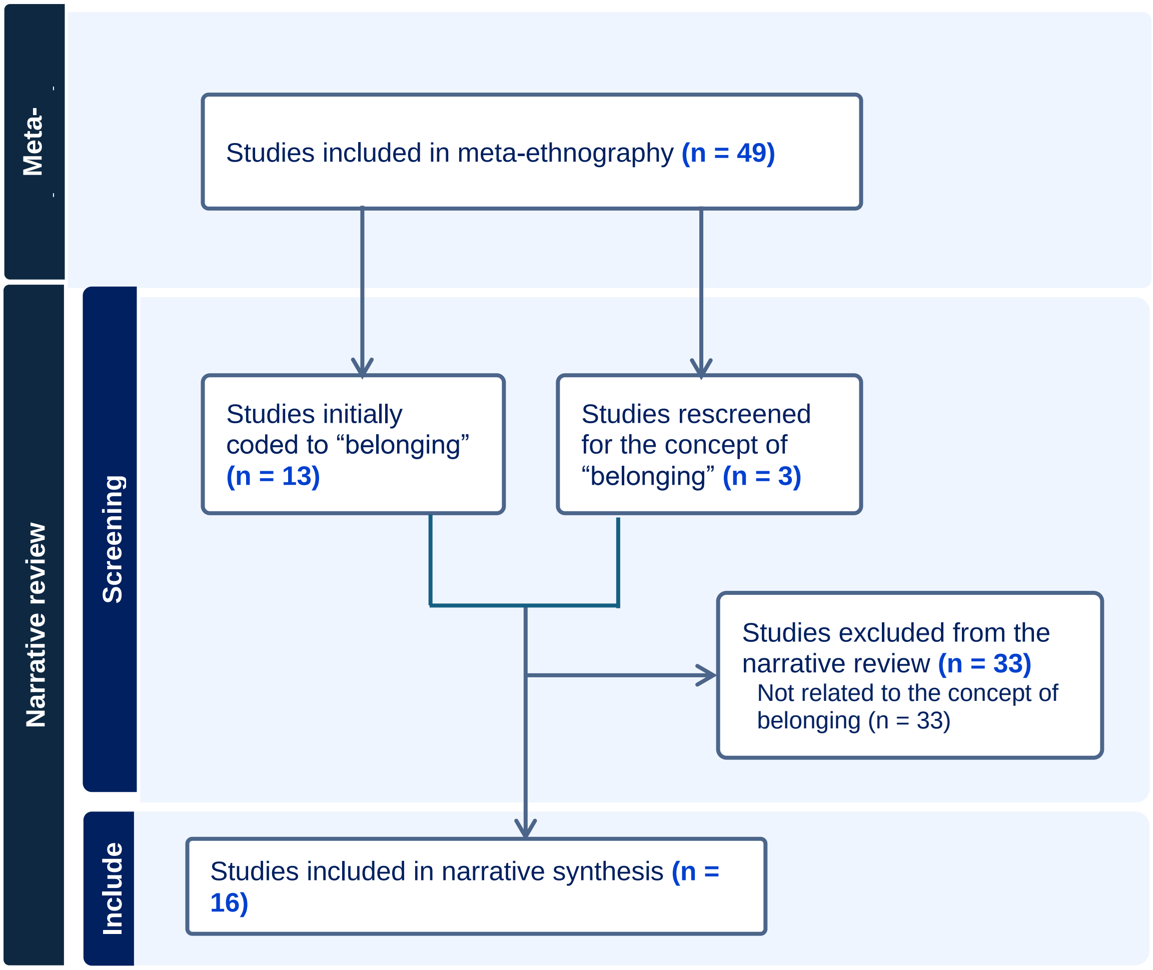

Supplement: Supplementary Material 2. — Derivation of the narrative review sample from the published meta-ethnography. [file pme-14-1-1873-s2.pdf]
