# Supplementary material for: The Struggle to Belong for Underrepresented Medical Students: A Narrative Review
Source: Perspect Med Educ. 2025 Nov 14;14(1):826–36. doi: 10.5334/pme.1873 (PMC12617423; doi:10.5334/pme.1873)
Supplement: Supplementary Material 3. — Characteristics of included studies. [file pme-14-1-1873-s3.pdf]

Supplementary Table 1: Characteristics of included studies

Author(s) & year, country	Participant characteristics	Reflexivity? Authors with lived experience?	Research aim	Methodology & analysis	Data collection	Summary of findings
Brosnan 2016, Australia	22 Indigenous; First in Family; Rural/Remote; mature medical students	No.<br><br>No noted authors with lived experience.	To draw on the theory of Bourdieu to explore FiF students' experiences at one Australian medical school, aiming to identify barriers and strategies	Qualitative study<br><br>Critical methodology<br><br>Conceptual framework: Bourdieu's forms of capital (social, economic and cultural)	Interviews	The absence of social capital (networks) was a barrier to connecting with fellow students and accessing placements. Financial challenges: expenses associated with medical school, juggling paid work with studying. 'Medical student' status brought new forms of cultural capital, a transition that was received with some ambivalence by participants and their social networks.
Bullock 2024, United States	16 'diverse' medical students: eight identified as women, two non-binary, six men, five from racially underrepresented groups, five LGBQ and three with a disability	In the methods the authors identity multiple diverse and intersecting identities. The first author constructs his identity as: "a gay Black man, physician and current post-graduate trainee"	To draw upon identity-salient experiences of medical students to describe a theory of identity safety.	Qualitative research Constructivist Grounded Theory	Interviews	Participants experienced identity threat through unwelcoming learning environments, feeling compelled to change their behaviour in inauthentic ways or sociopolitical threat. Participants characterised identity safety as the ability to exist as their authentic selves without feeling the need to monitor how others perceive their identities. Identity safety manifested when participants demonstrated agency to leverage their identities for patient care, when others upheld their personhood and saw them as unique individuals and when they felt they belonged in the learning environment.
Butler 2019, Canada	7 2SLGBTQIA+ medical students	In Methods, positionality of authors is mentioned.	to understand the experiences of TGNC medical students in	Grounded theory<br><br>Constructivist approach	Interviews	Main theme: navigating cisnormative medical culture

Author(s) & year, country	Participant characteristics	Reflexivity? Authors with lived experience?	Research aim	Methodology & analysis	Data collection	Summary of findings
		All authors have lived experience.	Canada. (Trans and gender nonconforming (TGNC))			Subthemes: culture and context; interactions with classmates, curriculum, policy, and administration; and gendered spaces.
Foreshew 2022, United Kingdom	4 medical students who self-identified as "less privileged" or from marginalised groups	In Methods section and throughout.<br><br>Authors have lived experience.	To ask medical students 'What is your experience of marginalisation at medical school?'<br><br>To openly explore intersectional experiences of marginalisation.	Action research<br><br>Bourdieu's theories re how social class hierarchies are reproduced in medical culture, healthcare and society	Comics-based workshop s and 1:1 interviews	Students' experience, feelings and ideas give us a source of knowledge to challenge classist, racist and sexist degradation widespread in medical culture. In particular, class elitism negatively impacted three women of working-class origins.
Isik 2021a, Netherlands	18 Ethnic minority medical students from years 1 to 6 with immigrant parents from: Afghanistan, Armenia, Egypt, Ghana, Philippines, Morocco, Nigeria, Russia, Syria, Turkey, Ukraine, and Uzbekistan	Methods, under heading "Reflexivity" refers to "the ethnic minority background of the main researcher"<br><br>Lead author has lived experience.	To gain insight into what support medical students from ethnic minorities need in their learning environment to mitigate experienced barriers, sustain their motivation and ultimately perform to their full potential.	Qualitative study<br><br>Constructivist, thematic analysis	Interviews	Students' negative experiences could be categorized as:<br>1. the effect of discrimination<br>2. lack of ethnic minority role models,<br>3. lack of belonging,<br>4. lack of a network,<br>5. differences and difficulties in cultural communication and language, and<br>6. examiner bias in clinical assessments.
Isik 2021b, Netherlands	26 Gender, ethnic, racial minority medical students	At the end of the methods. They mention it in the limitations again as potential taking the experiences for granted because of the team's diversity.<br><br>Lead author and one co-author have lived experience.	To investigate how medical students' ethnic identities and their intersection with other aspects of diversity relate to their motivation.	Qualitative study<br><br>Thematic analysis	Focus groups	Three main themes:<br>1. the role of autonomy in the formation of motivation, including students' own study choice and the role of their family;<br>2. interactions/'othering' in the learning environment, including feelings of not belonging; and<br>3. intersection of ethnic minority background and gender with being 'the other', based on ethnicity.

Author(s) & year, country	Participant characteristics	Reflexivity? Authors with lived experience?	Research aim	Methodology & analysis	Data collection	Summary of findings
Karasz 2023, United States	12 underrepresented in medicine Hispanic/Latinx, Black or Multiracial students	In the methods section they note that several authors experienced medical school as "minority students"	to investigate the experiences of the learning environment of underrepresented and non-underrepresented students.	Qualitative study	Interviews	Findings organized under three key headings related to perceptions of the learning environment: organizational (described a lack of adequate support and structure of the curriculum), social (included adversarial relationships with clinical supervisors and a sense of imposterism) and physical (included the value of dedicated safe spaces/rooms that enabled them to be themselves).
Ly 2024, United Kingdom	6 Black Asian Minoritised Ethnic (BAME) and Lesbian, Gay, Bisexual, Transgender, Queer (LGBTQ+) students	In Methods section both authors identify as BAME and the first author as LGBTQ+	to explore the experiences of BAME, LGBTQ+ medical students and how this affected their professional identity formation through an intersectional lens.	Qualitative phenomenographic study	Interviews	They identified six themes, categorised into three main areas: challenges to intersectionality, benefits to intersectionality and protective factors. Challenges to intersectionality included, 'The BAME identity interacting with the LGBTQ+identity', 'BAME background influencing LGBTQ+identity exploration' and 'no true safe spaces for BAME and LGBTQ+ students to be themselves'. Benefits to intersectionality included 'greater insight into the self' and protective factors included two themes of 'peer support networks' and 'visibility within the university and clinical environments.'
Morrison 2023, United Kingdom	20 medical students with "racially minoritised backgrounds"	In Methods section and then briefly in the discussion section under "strengths and	to identify experiences of racial microaggressions among RM medical students;	Qualitative study	Focus groups	Participants reported numerous racial microaggressions. These impacted directly and indirectly on their learning,

Author(s) & year, country	Participant characteristics	Reflexivity? Authors with lived experience?	Research aim	Methodology & analysis	Data collection	Summary of findings
		weaknesses.”<br><br>Lead author and three co-authors have lived experience.	to explore student perspectives on how their experiences of microaggressions impacted on their learning and performance; and to use the lens of the student participants to identify how medical schools can reduce racial microaggressions and build more inclusive learning environments.			performance and well-being. Students reported feeling uncomfortable and out of place in teaching sessions and clinical placements. They reported feeling invisible and ignored in placements and not being offered the same learning opportunities, leading to disengagement. Students described feelings of apprehension and having their ‘guards up’, particularly with new clinical placements, an additional burden.
Sivananthajothy 2023, Canada	16 Black; Disabled; 2SLGBTQ+; Gender minority medical students.	In Methods under heading “Strategies to promote rigour and reflexivity.”<br><br>Lead author and three co-authors have lived experience.	to explore how students from equity-deserving groups (EDGs) experience belonging during medical school, including those who are women, racialized, Indigenous, disabled, and 2SLGBTQ+.	Mixed methods; interviews were based on survey results re belonging, burnout, depression, impostor syndrome	Interviews	Participants described belonging as being able to exist as one’s “true self” while emphasizing feelings of acceptance, comfort, and safety as well as being valued and seen as an equal - yet described how routine experiences of “othering” inhibited a sense of belonging, often due to differences in social identity and structural privilege. Poor sense of belonging negatively affected learners’ wellbeing and career trajectory. We illuminate the range of psychological and professional consequences associated with diminished sense of belonging and highlight the need to expand traditional notions of equity, diversity, and inclusion to consider structural barriers to

Author(s) & year, country	Participant characteristics	Reflexivity? Authors with lived experience?	Research aim	Methodology & analysis	Data collection	Summary of findings
						belonging.
Southgate 2017, Australia	21 First in Family medical students	No.<br><br>No noted authors with lived experience.	To explore the research question: What are the experiences of FiF medical students in medical education and how do they understand their personal and professional journey through a high-status professional degree?	Qualitative study	Interviews	Students described getting to medical school 'the hard way'. Many felt like 'imposters', using self-deprecating language to highlight their lack of 'fit' in the privileged world of medicine. However, such language also reflected resistance to middle-class norms and served to create solidarity with community of origin, and, importantly, patients. Students' stories reflect a tactical refinement of self and incorporation of certain middle-class attributes, alongside an appreciation of the worth their 'difference' brings to the profession.
Strayhorn 2020, United States	5 Black men medical students	No.<br><br>No noted authors with lived experience.	to investigate the role of race (and racism) and sense of belonging for Black men in medical school, asking: How do Black men in medical school describe their experiences?	Qualitative study	Interviews	Race adversely affected students' academic and social experiences and diminished their sense of belonging in medical school. Consequently, they faced: difficulty connecting with White peers and faculty, racist stereotypes, and racial microaggressions that stigmatized them as "out of place," unqualified, or unusual.
Van Buuren 2021, Canada	16 2SLGBTQ+, Racialized, and/or lower social class First-year medical students	In Methods (Study design).<br><br>Authors have lived experience.	to understand how the first-year medical students' transition into medical school is influenced by their perceptions and experiences of diversity and inclusion during	Qualitative study, thematic analysis	Interviews	Participants highlighted the importance of social orientation during their transition into medical school and noted experiencing complex social pressures during this time. They shared

Author(s) & year, country	Participant characteristics	Reflexivity? Authors with lived experience?	Research aim	Methodology & analysis	Data collection	Summary of findings
			orientation.			how incoming students were introduced to the dominant medical professional identity during orientation. Participants noted tensions during this period, many of which revolved around the dominant identity and their past, present and future selves.
Walker 2020, United Kingdom	1 disabled medical student.	Intertwined throughout the writing, but formally within "Introduction to the authors" section.<br><br>Lead author has lived experience.	to explore the lived experiences of a UK medical student with dyspraxia within the current culture of UK medical education.	Collaborative Autoethnography	Collaborative ethnographic data (interviews, written reflections)	Findings are narrative in text, categorised by the following sub-headings: "My dyspraxia/being different"; "Pervasive emotional impact"; "Impact on studies and career"; "Determination"; "Coping strategies"; "The importance of others' reactions".
Wright 2023, Canada	17 First in Family medical students	In Methods section, right before Findings.<br><br>Authors have lived experience.	to explore, using a critically reflexive lens, the experiences of FiF students to better understand the ways in which the medical school environment can be exclusive and inequitable to underrepresented students.	Qualitative study; Bourdieu's theories and concepts were used as sensitizing concepts to explore the data.	Interviews	FiF students discussed the implicit messages they received about who belongs in medical school, challenges in shifting from their pre-medical lives to a medical identity and competing with peers for residency programs. They reflected on the advantages they perceived they had over their fellow students due to their less 'typical' social backgrounds.
Wyatt 2020, United States	14 Black medical students	In Methods.<br><br>Authors have lived experience.	to examine how URM students took active steps to negotiate their professional identity, considering the larger sociohistorical context surrounding minoritized individuals.	Qualitative study.	Interviews	URM students were aware of racist stereotypes and the potential for the medical community to view them negatively. In response, students employed identity cues and strategies to bring the community's perceptions in line with how they

Author(s) & year, country	Participant characteristics	Reflexivity? Authors with lived experience?	Research aim	Methodology & analysis	Data collection	Summary of findings
			Understanding these nuances in URM students' negotiation process of a professional identity will better inform medical schools interested in supporting the development of URM physicians.			perceived themselves—black and a physician. Specifically, students actively worked to integrate racial and professional identities by "giving back" to the African American community. Community-initiated mentoring from non-URM physicians helped to reify students' hope that they could have a racialized professional identity.
